# Prevalence of micronuclei in exfoliated uterine cervical cells from patients with risk factors for cervical cancer

**DOI:** 10.1590/S1516-31802008000600006

**Published:** 2008-11-06

**Authors:** Lízia Maria Franco dos Reis Campos, Francisca da Luz Dias, Lusânia Maria Greggi Antunes, Eddie Fernando Candido Murta

**Keywords:** Cells, Cervix uteri, Risk factors, Neoplasms, Micronucleus test, Células, Colo do útero, Fatores de risco, Câncer, Testes para micronúcleos

## Abstract

**CONTEXT AND OBJECTIVE:**

Pap smears are the most common and inexpensive screening method for cervical cancer. We analyzed micronucleus prevalence in exfoliated cervical mucosa cells, to investigate associations between increased numbers of micronuclei and risk factors for cervical cancer.

**DESIGN AND SETTING:**

Analytical cross-sectional study, at Instituto de Pesquisa em Oncologia (IPON).

**METHODS:**

Exfoliated cervical cells were obtained from 101 patients between September 2004 and November 2005. Patients’ ages, habits (passive or active smoking, alcoholism and numbers of sexual partners), age at first sexual intercourse, contraceptive methods used, histories of sexually transmitted diseases, use of hormone replacement therapy, numbers of pregnancies and abortions, inflammatory cytology and cervical intraepithelial neoplasia (CIN) were obtained. Cells were collected using Ayre spatulas, transferred to vials containing 0.9% saline solution for micronucleus tests and analyzed at 1000x magnification. The number of micronuclei in 1,000 epithelial cells per patient sample was counted.

**RESULTS:**

Comparisons between groups with active (7.9 ± 7.8) and passive (7.2 ± 10.6) smoking versus no smoking (3.7 ± 5.1); with/without alcoholism (7.8 ± 1.4 and 6.9 ± 10.1); with/without inflammatory cytology (10.7 ± 10.5 and 1.3 ± 1.7); and with CIN I, II and III and no CIN (respectively 4.3 ± 4.3, 10.6 ± 5.3, 22.7 ± 11.9 and 1.3 ± 1.4) found elevated micronucleus prevalence (P < 0.05).

**CONCLUSIONS:**

We concluded that the prevalence of micronuclei in exfoliated uterine cervical cells was greater in patients with one or more risk factors for uterine cervical cancer than in patients without risk factors.

## RESUMO

**CONTEXTO E OBJETIVO:**

O câncer do colo uterino é uma das mais freqüentes neoplasias na mulher. O exame de Papanicolaou é o método mais comum e econômico para rastreamento. As células esfoliativas epiteliais podem ser úteis para o monitoramento de pacientes expostas a fatores de risco para o câncer. O objetivo foi analisar a prevalência de micronúcleos em células esfoliativas da mucosa cervical uterina e associar com fatores de risco para o câncer de colo uterino.

**TIPO DE ESTUDO E LOCAL:**

Estudo transversal analítico, no Instituto de Pesquisa em Oncologia (IPON).

**MÉTODOS:**

Células esfoliativas do colo uterino foram obtidas de 101 pacientes ambulatoriais entre setembro/2004 e novembro/2005. As células foram coletadas usando espátula de Ayre e transferidas para um tubo de ensaio com soro fisiológico 0,9% para o teste do micronúcleo. Informações obtidas das pacientes foram: idade, hábitos (fumo e número de parceiros sexuais), métodos contraceptivos, história de doença sexualmente transmissível e uso de terapia hormonal. Células foram analisadas com magnificação de 1000 X e os micronúcleos contados em 1.000 células epiteliais por paciente.

**RESULTADOS:**

A comparação do grupo de pacientes fumantes ativas (7,9 ± 7,8) e passivas (7,2 ± 10,6) *versus* não fumantes (3,7 ± 5,1); alcoolismo e não alcoolismo (7,8 ± 1,4 e 6,9 ± 10,1); citologia inflamatória e citologia normal (10,7 ± 10,5 e 1,3 ± 1,7); neoplasia intraepitelial cervical (NIC) I, II e III e a ausência de NIC, respectivamente, (4,3 ± 4,3; 10,6 ± 5,3; 22,7 ± 11,9 e 1.3 ± 1.4) mostrou maior prevalência de micronúcleos (P < 0,05).

**CONCLUSÕES:**

A prevalência de micronúcleo nas células esfoliativas do colo uterino foi maior no grupo de pacientes com pelo menos um dos fatores de risco para câncer do colo uterino do que no grupo controle (sem fatores de risco).

## INTRODUCTION

Cervical cancer is one of the most frequent female cancers. The estimated worldwide incidence of cervical cancer is approximately 500,000 new cases per year, and the overall five-year survival rate is in the range of 44 to 66% for all clinical stages. Pap smears are the most common and inexpensive method of screening for cervical cancer.^1,2^

Since most cancers arise in epithelial tissues, exfoliated epithelial cells may be particularly useful for monitoring patients who are exposed to risk factors.^3^ Epidemiological evidence indicates that in most cervical cancer patients, squamous cell carcinoma is the predominant histological type. This carcinoma results from progression of preinvasive cervical intraepithelial neoplasia (CIN) grade I to CIN III.^4^ The evolution of CIN I to III is accompanied by increased genetic instability or mutability, such as losses or gains of chromosomes or fragments of chromosomes.^5,6^ Progression to advanced-stage cervical carcinoma is characterized by a recurrent pattern of chromosomal rearrangements. The pattern of abnormalities varies greatly between malignancies, ranging from simple balanced rearrangements to complex abnormalities affecting both chromosome structure and number.^7^

In addition to genetic factors, various environmental factors have also been implicated in the neoplastic process. Among these, human papillomavirus (HPV) infection and smoking have been cited. HPV infection is one of the most common sexually transmitted diseases and is associated with a higher risk of cervical cancer.^8^ Extensive screening programs and the development of safe and effective vaccines against HPV would diminish mortality and morbidity from this disease, which has been reported to affect poor women disproportionately.^8,9^

Behavioral risk factors such as smoking indirectly influence the manifestation of cervical cancer and thereby accelerate the tumor progression induced by HPV. Smoking may contribute towards the development of cervical cancer through direct exposure of the DNA of epithelial cells to nicotine and cotinine, or through reactions with the metabolic products from the smoke, such as aromatic polycyclic hydrocarbons and aromatic amines.^10,11^ According to Weiderpass et al.,^12^ alcohol consumption may have an indirect influence on the development of cervical cancer, by triggering malignant transformation of HPV lesions.

Carcinogens affect cells by altering genetic material and thus causing instability. Chromosomal instability manifested by increased aneuploidy and structural chromosomal aberrations is believed to play a critical role in the intermediate to late stages of the development of cervical malignancies.^13^ Chromosomes or chromosome segments that fail to be incorporated into nuclei during cell division configure micronuclei. Thus, micronuclei represent a measure of both chromosome breakage and chromosome loss, and can function as a sensitive indicator of genetic damage.^14^

## OBJECTIVE

The purpose of this study was to analyze the prevalence of micronuclei in exfoliated cells from the cervical mucosa in order to investigate associations between increased numbers of micronuclei and risk factors for cervical cancer, including smoking, large numbers of sexual partners, histories of sexually transmitted diseases (STDs), presence of infectious agent for vaginitis, and diagnoses of cervical intraepithelial neoplasia (CIN).

## MATERIALS AND METHODS

### Patient and control characteristics

Exfoliated cervical cells were obtained from 101 patients who were treated at the Gynecology and Obstetrics outpatient service of the teaching hospital of Universidade Federal do Triângulo Mineiro (UFTM), Uberaba, Minas Gerais, Brazil, between September 2004 and November 2005. All these patients had previously undergone the Papanicolaou test and colposcopy, respectively to detect likely infectious agents for vaginitis and preinvasive or invasive malignant lesions of the uterine cervix. All the patients underwent video colposcopy, and biopsies were performed in the cases where test abnormalities were present, in accordance with the Rome recommendations.^15^ The patients’ ages ranged from 36 to 82 years, with a mean of 58.2 ± 11.5 years (mean ± standard deviation, SD).

Out of the total of 101 patients, 12 did not present any of the risk factors and were therefore considered to be controls. The other patients were divided into groups according to the factors considered in this study: numbers of partners, pregnancies and abortions, smoking, alcoholism and presence of agents for vaginitis infection. A Specific ELISA (enzyme linked immunosorbent assay) was used to investigate the serological presence of the human immunodeficiency virus (HIV), and positive findings were confirmed by western blot and indirect immunofluorescence.

After the patients signed an informed consent form, cells were collected. The study was approved by the Research Ethics Committee of UFTM (protocol no. 528/2005). All the participants answered personal questions regarding their lifestyles. Information on the patients’ ages, habits (smoking, drug use and numbers of sexual partners), contraceptive methods used, histories of STDs and use of hormone replacement therapy was obtained using a modified version of the questionnaire of the Commission for Protection against Environmental Mutagens and Carcinogens.^16^

### Cervical cell collection and preparation

Exfoliated cervical cells were collected using Ayre spatulas and were transferred to vials containing 0.9% physiological serum for micronucleus tests. The material was centrifuged and the supernatant was discarded, leaving the exfoliated cells in the pellet. The cells were fixed for 20 minutes using 1 ml of a methanol-acetic acid solution (3:1). Drops of the material were placed on cold damp slides and allowed to dry. Samples were stained using 4% Giemsa for 12 minutes.

The cytological criteria were used as previously described.^17-19^ The clue cells were squamous cells covered with coccobacilli that presented smudged cytoplasmic borders. *Candida sp.* was diagnosed through the presence of pseudohyphae that stained weakly with hematoxylin-eosin and/or small spores (diameters of 2-4 mm) that stained pale pink. The cytological diagnosis for possible HPV infection was based on the morphological criteria of Schneider et al.^20^

### Cytogenetic analysis of micronuclei

**Figure 1:**
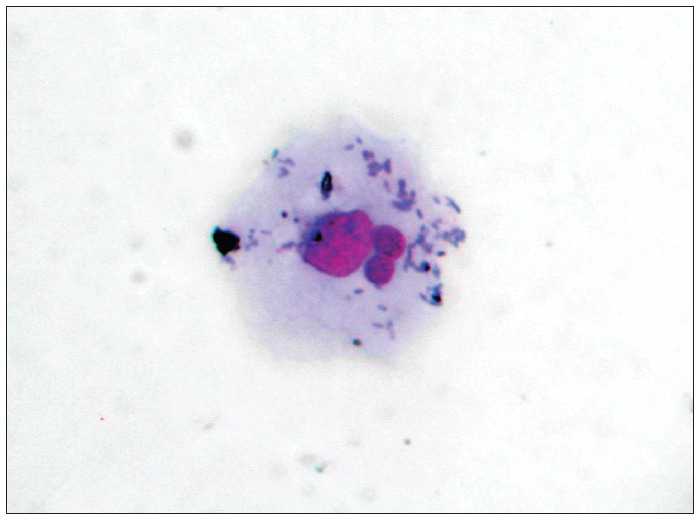
Exfoliated cervical cell with two micronuclei (magnification 1000 X).

The slides were analyzed using an optical microscope at a magnification of 1000 x (objective = 100 x with eyepiece = 10 x), and 1,000 epithelial cells were counted per microscope field. Within the samples, only cells that were separate, without overlapping or folds, were analyzed. Micronuclei were counted if the structures had a regular border and were located inside the cytoplasm, with an intensity of staining less than or equal to that of the main nucleus and a size less than two-thirds of the size of the main nucleus (Figure 1). The frequency of micronuclei was obtained as the ratio between the number of micronuclei and the total number of cells analyzed, multiplied by 100.

### Statistical analysis

Statistical analyses were computed using GraphPad InStat 3 (GraphPad Software, Inc). All variables were subjected to the Kolmogorov-Smirnov test to determine whether there was normal distribution. The data with normal distribution were then analyzed using the parametric Kruskal-Wallis method and the Dunn multiple comparisons test. The data that did not present normal distribution were analyzed using the nonparametric Mann-Whitney method. The significance level considered for all the tests was P < 0.05.

## RESULTS

The frequency of micronuclei in cervical samples and the presence or absence of cervical cancer risk factors are shown in Table 1. These data showed a nonsignificant trend between low frequency of micronuclei and absence of putative risk factors such as age, age at first sexual intercourse, number of partners, use of oral contraceptives and numbers of pregnancies and abortions. Smoking and alcohol consumption each significantly increased the frequency of micronuclei, in relation to the groups that did not present these factors. However, no difference was observed between the active and passive smoking groups. The factors with the greatest influence on the frequency of micronuclei were inflammation and the presence of CIN (P < 0.001), in comparison with groups without these factors. It was also found that infectious agents for vaginitis, such as *Candida sp.* and *Gardnerella vaginalis* (i.e. presence of clue cells), and HIV-positive serology also affected micronucleus frequency, with significantly higher levels (P < 0.05) in positive samples than in the groups that had no infection. Since smoking influenced the frequency of micronuclei, an analysis comparing the nonsmoking women in the control group with those in the active and passive smoking group was performed.

**Table 1:** Micronucleus frequency in relation to risk factors for cervical cancer

Factor	Groups defined	Sample (n)	Micronucleus frequency (mean ± SD)	P-value
**Control**		12	3.70 ± 5.1	
**HPV** *		57	11.01 ± 1.08	0.04
**Age (years)** *	≤ 35 years	59	6.80 ± 8.80	0.486
	> 35 years	42	8.00 ± 10.30	
**Age at first sexual intercourse** *	≤ 16 years	56	6.70 ± 9.70	0.299
	> 16 years	45	8.10 ± 9.20	
Partner*,†	1	19	5.40 ± 8.20	0.328
	> 1	82	7.70 ± 9.70	
**Oral contraceptive**	Yes	55	6.10 ± 8.30	0.109
	No	46	8.70 ± 10.60	
**Pregnancy**	0	23	4.40 ± 6.70	0.093
	1 to 3	55	7.20 ± 8.90	
	> 3	23	10.40 ± 12.10	
**Abortion** *	Yes	31	9.70 ± 10.80	0.090
	No	70	6.20 ± 8.70	
Active smoker*,†	Yes	47	7.90 ± 7.80	0.006
	No	54	5.10 ± 10.00	
Passive Smoker*,†	Yes	36	7.20 ± 10.60	0.038
	No	65	5.90 ± 4.80	
Alcoholic*,†	Yes	31	7.80 ± 1.40	0.012
	No	70	6.90 ± 10.10	
Cytology with inflammation*,†	Yes	65	10.70 ± 10.50	0.0001
	No	36	1.20 ± 1.70	
CIN*,†	Yes	56	11.00 ± 10.40	0.0001
	No	35	1.30 ± 1.40	
**STD** *	Yes	51	6.70 ± 8.90	0.508
	No	50	7.90 ± 10.60	

* *P < 0.05, compared with control;*

† *P < 0.05, compared within the same group; STD = sexually transmitted disease; CIN = cervical intraepithelial neoplasia; SD = standard deviation; HPV= human papillomavirus.*

The presence of cytological signs of HPV infection increased the frequency of micronuclei among the infected women (Table 2). The patient samples were divided into two groups (with and without cytological signs of HPV infection), and a stratified analysis in relation to the other risk factors for cervical cancer was performed. The risk factors combined with HPV infection resulted in statistically significant differences in micronucleus frequency between the group in question and the groups that did not present HPV. Furthermore, vaginal and cervical inflammation was associated with very significantly increased frequency of micronuclei. Even when inflammation intensity (mild, moderate or severe) was taken into account in the analysis, the frequency of micronuclei was significantly greater in subjects with inflammation than in controls without inflammation (Table 3).

**Table 2:** Comparison of mean frequencies of micronuclei in women with and without HPV infection, in relation to risk factors for cervical cancer

Factor	HPV		No HPV	
	n	Micronucleus frequency (mean ± SD)	n	Micronucleus frequency (mean ± SD)
**Alcoholic** *	25	9.6 ± 8.1	6	2.5 ± 1.1
**Smoker** *	32	10.0 ± 7.8	15	1.7 ± 1.8
**Infectious agents for vaginitis** *	22	9.7 ± 8.2	9	4.0 ± 4.4
**Cytology with inflammation**	47	13.2 ± 10.7	18	10.7 ± 1.05
**CIN** *	53	14.2 ± 18.9	3	4.6 ± 4.9
**Oral contraceptive** *	30	9.8 ± 9.6	25	1.7 ± 2.2

* *P < 0.0001, for all data in comparison with no HPV; HPV = human papillomavirus; CIN = cervical intraepithelial neoplasia.*

**Table 3:** Comparison of the mean frequencies of micronuclei in the cytology with inflammation and smoking groups compared to controls

Factor	Sample (n)	Micronucleus frequency (mean ± SD)
**No inflammation**	**36**	**1.2** ± **1.7**
**Inflammation**		
Mild*	**20**	**5.5** ± **4.9**
Moderate*	**27**	**9.7** ± **8.5**
Severe*	**18**	**17.9** ± **13.0**
**Non-smoking**	**12**	**3.7** ± **5.11**
**Passive smoking** †	**36**	**7.2** ± **10.60**
**Active smoking** †	**47**	**7.9** ± **7.80**

* *P < 0.001 versus no inflammation;*

† *P < 0.05 versus non-smoking; SD = standard deviation.*

We observed (Table 4) that CIN correlated with increasing numbers of micronuclei. During the process of chromosomal damage, more than one micronucleus may be found within cells. Most of the cells observed in this study had one micronucleus. Cells with two micronuclei were most frequently observed in women who had had more than three pregnancies. The groups with more than three pregnancies, CIN, cytological signs of HPV infection and inflammation were the only subjects that presented more than three micronuclei per cell.

**Table 4:** Comparison between women without cervical lesions and women with different degrees of cervical intraepithelial neoplasia (CIN), in relation to the frequency of micronuclei

Factor	Sample (n)	Micronucleus frequency (mean ± SD)
No lesion	35	1.3 ± 1.4
Metaplasia*	10	7.2 ± 9.6
CIN I†	25	4.3 ± 4.3
CIN II*	16	10.6 ± 5.3
CIN III*	15	22.7 ± 11.9

* *P < 0.001 and*

† *P < 0.05 versus no lesion; SD = standard deviation.*

## DISCUSSION

In this study, we observed that 88% of the women had some type of risk factor for cervical cancer. Of these, 64% showed increased micronucleus frequency in exfoliated cervical cells. Young adult women were more vulnerable to risk factors, since they present a cervical transformation zone located in the ectocervix.^21^ Nonetheless, our results did not demonstrate any influence from age, age at first sexual intercourse or number of partners, with regard to micronucleus formation. However, the number of pregnancies and abortions increased the frequency of micronuclei. Prolonged exposure to hormones has been reported to be a strong risk factor for progression of intraepithelial neoplasia, since steroid hormones may facilitate the transforming activity of HPV.^22^ The use of oral contraceptives seems to increase the transforming activity of HPV oncogenes and interfere with the efficient resolution of lesions caused by this virus in the cervix among young women.^23^ Although use of oral contraceptives did not affect the expression of HPV activity, women who were both HPV-infected and oral contraceptive users presented significantly increased frequency of micronuclei, thus suggesting that the use of these steroid hormones might boost the oncogenicity of HPV infection.^24,25^

In the present study, we observed greater numbers of micronuclei in specimens from women who were active or passive smokers, relative to control specimens. These results corroborate those of Cerqueira et al.,^26^ who found greater numbers of micronuclei in exfoliated cervical cells in women who smoked than in those who did not. Among patients without lesions (both smokers and nonsmokers), the frequency of micronuclei was lower than among those who had some type of abnormal pathological condition. Among nonsmokers, the frequency of micronuclei was higher in patients with lesions than in those who were cytologically normal. Among smokers, the frequency of micronuclei was high, even in women with a low degree of inflammation. In an epidemiological study, Matsumoto et al.^27^ showed that smoking and *Chlamydia* infection were cofactors for CIN progression. Passive smokers were found to have the same risk of developing CIN as did active smokers.^28^ Two main mechanisms have been suggested through which smoking may contribute towards cervical carcinogenesis: one involves direct exposure of the deoxyribonucleic acid (DNA) in cervical epithelial cells to nicotine and cotinine, and the other involves exposure to metabolic products resulting from the reactions of other components of cigarettes such as aromatic polycyclic hydrocarbons and aromatic amines.^11,29^ Other mechanisms that may explain smoking-related carcinogenesis include abnormalities in the peripheral immune system of smokers, such as elevated numbers of cytotoxic/suppressor T lymphocytes, diminished numbers of helper T lymphocytes, suppression of T lymphocyte activity, significantly decreased numbers of natural killer lymphocytes and low levels of immunoglobulins other than immunoglobulin E (IgE).^30^ These effects may result from decreased numbers of Langerhans cells in the cervix of women who smoke.^31^

Women who consume alcohol are considered to present a high and progressive risk of developing *in situ* and invasive cervical and vaginal cancer. Epidemiological data have suggested a direct link between alcoholism and lifestyle factors such as promiscuity, smoking, use of hormonal contraceptives and dietary deficiencies.^12^ In the data obtained from the present study, significantly increased frequency of micronuclei was observed among the women who consumed alcohol.

Our results showed that women presenting inflammation had significantly greater numbers of micronuclei than did those without inflammation or those in the control group. Another important result was that the progressive increase in inflammation severity was directly proportional to the observed increase in micronucleus numbers. We observed a strong association between high numbers of micronuclei and the presence of inflammation with concomitant HPV infection, in relation to those without HPV infection. This observation may suggest that the presence of inflammation among women with HPV infection increases the genetic damage in cervical epithelial cells. The observation that only a small minority of the lesions resulting from HPV infection progressed to invasive cancer led Nishimura et al.^32^ to suggest that additional events were necessary for malignant cellular transformation.

In evaluating micronuclei in relation to the presence of CIN, we observed that greater numbers of micronuclei were seen in women with progressive increases in the severity of CIN (CIN I < CIN II < CIN III), compared with controls. This evidence corroborates the importance of the micronucleus test as a biomarker for malignancy. A study by Guzmán et al.^4^ showed an association between lesion severity and micronucleus frequency in epithelial cells, which contributes towards validating micronucleus frequency as a possible biomarker for cancer risk.

Women with STDs presented significantly higher frequencies of micronuclei than did the control group. Fischer observed that *Chlamydia* infection could cause cervical hypertrophy in women with or without CIN or carcinoma.^33^ However, concomitant infection by *Chlamydia* and HPV increased the expression of Ki67 in the epithelium. Moreover, *Chlamydia* infection has been shown to increase HPV-16 activity, possibly explaining variations in the HPV mechanism for cervical carcinogenesis.^33,34^ In a study based solely on cytological criteria, there was an association between *G. vaginalis* and HPV infection.^35^ Two other studies addressed the matter.^36,37^ One of them^36^ showed that, compared with the group of pregnant women without HPV infection, those with HPV infection had a significantly higher percentages of bacterial vaginosis (BV) (53.8 versus 15.4%; P = 0.007) and *Chlamydia trachomatis* (34.6 versus 7.7%; P = 0.039). No cases of *Neisseria gonorrhoeae* were diagnosed. All cases of *C. trachomatis* and BV had high-risk HPV.^36^ The other manuscript showed that there were higher frequencies of BV and HPV in patients with atypical squamous cells of undetermined significance than in patients with normal cytology.^37^

By most accounts, the oncogenic mechanism of HPV is well understood. Following infection by HPV, oncoproteins from the virus integrate with the tumor suppressor proteins p53 and pRb, which in turn alter the function of oncoproteins, thereby resulting in uncontrolled transcription activity, abnormal DNA replication and cell division, hence leading to tumor formation.^38^ Our results showed that the women with HPV infection had an elevated frequency of micronuclei in relation to the control group. In our study, HPV status was compared with a variety of risk factors for cervical cancer and the results were concordant with the literature.

There are three mechanisms that may contribute towards the formation of micronuclei: metabolic stress caused by tumor growth, clastogenic products released from tumor cells and the presence of HPV.^39^ Chromosomal instability, particularly in chromosomes 1, 3, 5, 11 and 17, is associated with the development of cervical carcinoma.^40^ The results presented in this study demonstrated that the presence of micronuclei correlated with malignancy, given that the results were significant in relation to various risk factors for cervical cancer. Micronuclei are indicative of numerical and/or structural chromosome aberrations during cell mitosis. Other authors have used the micronucleus test as a biomarker for chromosome instability and malignancy, observing higher frequencies of micronucleated cells among cancer patients than among healthy individuals.^41,42^ The presence of micronuclei has been considered to be a very useful biomarker for detecting malignant cervical uterine carcinomas.^39^ According to Bonassi et al.,^43^ several studies have confirmed the presence of micronuclei in different cell types, thus suggesting that micronuclei may be a morphological marker that may be useful for predicting several types of cancer risk. Furthermore, the ease and low cost of this method may allow further development of the micronucleus test as a prognostic indicator during the planning and validation of programs for cancer monitoring and prevention. Furthermore, the micronucleus test has been considered to be a very useful biomarker for detecting malignancy in the uterine cervix, with regard to other factors like surgical margins and the numbers of mitoses and methylated genes, for predicting recurrence of CIN III.^44-46^

The number of micronuclei correlates with the severity of genetic damage. Cells containing several micronuclei present greater genetic damage than do cells that present only one micronucleus. The data obtained in this study suggest that factors such as HPV infection, number of pregnancies, CIN and inflammation are more clastogenic because they increase the frequency of cells containing more than three micronuclei. This could explain the number of micronuclei found in metaplasia. Taken together, comparison in our study between patients with and without risk factors for cervical cancer showed that there was a significant difference, thus suggesting that micronuclei may be a valid biomarker for cancer risk.

## CONCLUSIONS

We conclude that the prevalence of micronuclei in exfoliated uterine cervical cells was greater in the patients with one or more risk factors for uterine cervical cancer than in the patients without risk factors.
